# Pure Water

**Published:** 1897-06

**Authors:** 


### Pure Water.

Just what a pure supply means is hardly open to much debate and may be summed up in five propositions, as follows:

1. That the water supply of any city or village should not in any possible way be liable to pollution or contamination from the sewage of any other community.
2. That the sewage of a city should not be emptied into any water-course not having a current of three to five miles per hour, and then the sewage entrance should be at a distance one mile or more from the intake.
3. When the water supply of any city or village is a navigable stream, the water should be sand-filtered before being pumped into the city reservoirs of water-mains.
4. That for ordinary drinking purposes, the water should not be taken in its primitive or raw state, but be either filtered, boiled, or distilled and aerated.
5. That not only chemical, but bacteriological, examinations of the water should be made, at least once weekly, to determine its character as a safe or dangerous water for domestic use, and where contamination is shown to exist, the services of an engineer be enlisted to detect, if possible, the cause and origin of such contamination.—*Buffalo Med. Jour.*

